# Preliminary Statistics Issued by the Census Bureau

**Published:** 1911-07

**Authors:** 


### Preliminary Statistics Issued by the Census Bureau.

WASHINGTON, D. C., July 1, 1911.

Acting Census Director Falkner has received from Dr. J. A. Hill, chief statistician for revision and results in the Census Bureau, a preliminary count of the population in institutions comprising prisons, institutions for juvenile delinquents, alms houses and institutions for the insane and feeble-minded. The enumeration includes the number present in the institutions on January 1, 1910, and the numbers admitted and discharged during the year 1910. A few institutions still remain to be heard from.

According to this preliminary count the prison population on January 1, 1910, was 109,311; the admissions or commitments to prisons during the year 1910 were 462,530, and the number of prisoners discharged during that year on account of expiration of sentence or other reasons, including also deaths, was 458,996.

The last previous census of prisoners was taken June 30, 1904, and at that time the prison population was 81,772, and the admissions or commitments during that year 149,691. These figures, however, are not comparable with those for the year 1910, for the reason that the 1910 enumeration included cases of imprisonment for non-payment of fine, while the census of 1904 did not include such cases.

---

BECKVILLE, TEXAS, June 21, 1911.

*Dr. F. E. Daniel, Austin, Texas.*

MY DEAR DOCTOR: I again enclose check for JOURNAL. It seems to get better and dearer to me as I grow old. My first copy came to me just as my old friend, E. S. Gailliard, so kind, yet fearless and bold, when occasion required, was passing away. Your editorials and comments so much remind me of him.

M. D. STERRETT.

CHIHUAHUA, MEXICO, July 5, 1911.

*Dr. F. E. Daniel, Austin, Texas.*

DEAR DOCTOR: I enclose bank draft on El Paso for $1.00 to pay my subscription to the "Red Back" to January, 1912, as per enclosed slip.

I enjoy reading your breezy magazine and admire you for your stiff "backbone." The "Red Back" has a field of its own that is not cultivated by any other medical magazine that I ever saw, and it is well worth the price.

Yours fraternally,

HOWARD D. EATON, M. D.

---

CHICAGO, ILL., July 3, 1911.

*To the Texas Medical Journal, Austin, Texas:*

Kindly take notice that the book published by one A. V. Harmon and one A. J. Jackman, under the name of "Large Fees and How to Get Them" of which I am alleged to be a joint author, is, so far as the use of my name is concerned, a forgery. All persons selling or circulating same, or advertising or reviewing the book in connection with my name, do it at the risk of legal complications. Other journals please copy, and oblige,

G. FRANK LYDSTON.

---

When performing lateral anastomosis after intestinal resection, make the opening reasonably near the closed ends. Long blind pouches may give trouble.—*American Journal of Surgery.*

